# Understanding the Role of Social Negativity in Perceived Life Course Impact and Mental Health Among Women with Endometriosis

**DOI:** 10.3390/jcm14134761

**Published:** 2025-07-05

**Authors:** Chen Zarecki, Carmit Satran, Anis Kaldawy, Riki Tesler, Shiran Bord

**Affiliations:** 1 Department of Health Systems Management, Faculty of Health Sciences, Ariel University, Kiryat Hamada 3, Ariel 40700, Israel; riki.tesler@gmail.com; 2 Department of Health Systems Management, The Max Stern Yezreel Valley College, Emek Yezreel 1930600, Israel; shiranb@yvc.ac.il; 3 Department of Nursing, The Max Stern Yezreel Valley College, Emek Yezreel 1930600, Israel; carmits@yvc.ac.il; 4 Clalit Health Services, Haifa and Western Galilee District, Hameyasdim 7, Kiryat Bialik 2706716, Israel; aniskaldawy@gmail.com

**Keywords:** endometriosis, life course impact, social support, social negativity, mental health

## Abstract

**Background**: Endometriosis is a chronic, inflammatory, estrogen-dependent gynecological disease in which endometrial-like tissue grows in areas outside the uterus. This condition may significantly influence women’s life course and mental health. Personal, behavioral, social, and environmental factors play a crucial role in predicting these outcomes. The current study aimed to compare the Perceived Life Course Impact (PLCI) and mental health of women with Endometriosis to those without the disease, as well as to explore the factors associated with PLCI and mental health. **Methods**: This cross-sectional study surveyed 543 Israeli women (270 with Endometriosis, 273 without). Participants completed a validated questionnaire assessing perceptions of life course impact in several life domains (intimacy and relationships, employment, education) and mental health. Multiple regression analyses were used to identify factors associated with PLCI and mental health among participants. **Results**: Women with Endometriosis reported a significantly greater negative perceived impact on intimacy and relationships, employment, and education, with poorer mental health, as compared to the control group. Women with Endometriosis also experienced higher social negativity and lower social support. Multiple regression analyses indicated social negativity as a strong predictor of negative PLCI. Additionally, menstrual pain, social support, and healthcare accessibility were found to be significant predictors of both PLCI and mental health. **Conclusions**: Comprehensive care for women with Endometriosis requires a multidisciplinary approach, with interventions focused on improving healthcare accessibility, enhancing social support networks, and mitigating social negativity within interpersonal environments.

## 1. Introduction

Endometriosis is a chronic, inflammatory, estrogen-dependent gynecological disease in which endometrial-like tissue grows in areas outside the uterus. Endometriosis symptoms vary in severity and manifestation, with different effects on patients’ lives [1]. Chronic pelvic pain, painful menstruation, painful urination, pain during intimate relations (dyspareunia), painful bowel movements, weakness, and infertility are the most common symptoms [2].

Since the clinical representation of Endometriosis varies, it can be challenging to diagnose based solely on symptoms [2]. In recent years, advancements in imaging modalities have redefined the gold standard for diagnosing Endometriosis, shifting from visual identification and confirmation of pathology, typically attained through laparoscopic surgery, to relying on imaging techniques, such as ultrasound (US) or magnetic resonance imaging (MRI) [2].

The prevalence of the disease in the general population is not known precisely, and reports indicate a wide range. Researchers have proposed several explanations for the variability found in studies: the diverse presentation of symptoms, diagnostic delays, and the lack of reliable non-surgical diagnostic tools [3]. Another explanation is that the high estimated prevalence is likely due to most studies being conducted on high-risk samples of women who frequently visit hospitals and gynecologic clinics [4,5].

A review by Harder et al. [3] examined the prevalence of Endometriosis from 2000 to 2023, encompassing a total of 103 papers from 36 countries. The review indicates a substantial disparity across four sources: (a) Health insurance data reports reveal the lowest prevalence of the disease, at 0.7%. This source includes data from Hungary at 0.19% [6], South Korea at 0.21% [7], and Italy at 2% [8]. (b) The pooled prevalence found in clinical data was 6.8% from 16 different countries; for example, among women aged 15–55, the prevalence in Denmark is 1.63% [9]; Iceland, 3.6% [10]; Canada, 10.91% [11]; Saudi Arabia, 11.1% [12]; and Jordan, 13.7% [13]. (c) The third source, based on population-based surveys, found the pooled prevalence to be 6.56%. Among women aged 18–50, for instance, the prevalence in the USA is 6.1% [14]; Australia, 3.4% [15]; Canada, 7.0% [16]; and Turkey, 18.3% [17]. (d) The highest prevalence, found in studies of symptomatic women, stands at 13% [3]. For example, among women aged 18–55, the prevalence in the USA was found to be 10.2% [18] and 39.4% [19], while in Australia, it was 6.9% [20]. One of the studies mentioned in the review [3] was conducted by Eisenberg et al. [4] among women aged 15–55 insured by Maccabi Health Services in Israel. The results show a prevalence found in the health insurance reports standing at 1.08%.

Endometriosis Over Women’s Life Course

Considering the prevalence patterns described above, understanding the profound effects of Endometriosis on women’s lives over time is critical, as Endometriosis symptoms significantly impact women’s physical and mental health, intimate relationships, reproductive health and well-being, and may influence their life course [2,21]. The ‘life-course impact’ approach examines how a disease, along with its physical, social, mental, and/or emotional consequences, and the experience of living with the disease influence an individual’s life course in terms of well-being and goal achievement. When applied to the study of chronic diseases, this approach views disease as potentially having cumulative negative effects on an individual’s life course [22,23]. However, it can also lead to positive outcomes, such as the development of coping skills and the receipt of social and family support [22,23]. Moreover, life course research explains that each life stage is shaped by its historical context, life events, and relationships with others [24,25], as well as subjective perceptions [26].

In this study, we measure Perceived Life Course Impact (PLCI) by examining participants’ reports on their subjective life course impact. Previous research [27,28] has demonstrated that individuals’ perceptions of their health status can accurately reflect their actual condition. Therefore, we assume that PLCI effectively represents the objective impact on the participants’ life course.

Missmer et al. [23] reviewed relevant studies from 2009 to 2019 and found that Endometriosis and its symptoms impact women’s lives at major milestones in life: education, career, and intimate relationships. The impact on education begins in childhood and continues even at a mature age. Two-thirds of women reported they had to take time off school and had difficulty focusing and doing schoolwork due to the disease. Furthermore, Endometriosis symptoms can affect the choice of academic discipline and the decision whether to continue to higher education, with many losing educational opportunities due to their pain and symptoms [23,29].

Women’s employment and career choices are also affected by Endometriosis. In the abovementioned review article, more than 15 studies indicated decreased productivity, delay in professional development, and lower wages [23]. According to Nnoaham et al. [30], Endometriosis was associated with an average loss of 11 h of work productivity per week. Women aged 25–34 who participated in Moradi et al.’s [29] study said that having Endometriosis made them less productive at work, choose part-time work, and/or give up their favorite jobs. These findings were also found in other studies, indicating that many women with Endometriosis could not manage full-time employment due to Endometriosis symptoms [31]. Fourquet et al. [32] studied women with self-reported and surgical diagnoses and found that 40% believed their professional growth had been directly and negatively related to the disease. The review by Missmer et al. [23] also indicated that women with Endometriosis tended to opt for less stressful and thus less meaningful jobs or even chose not to work.

It is essential to recognize the Perceived Life Course Impact (PLCI) of Endometriosis and its symptoms on women’s intimate relationships [21]. According to a cross-sectional study by De Graaff et al. [33], half of the women surveyed reported that Endometriosis had affected their intimate relationships, as well as reporting two to three times more pain during intercourse, with significantly higher VAS (Visual Analogue Scale) pain scores as compared to women without Endometriosis. Symptoms such as dyspareunia (pain during intercourse) may cause negative consequences to women’s self-esteem and intimate relationships [21,34,35]. Other Endometriosis symptoms, such as chronic pelvic pain and fatigue [36], as well as depression and anxiety [37], may also harm women’s sexuality [38].

Family planning represents a significant milestone in a woman’s life [23]. Infertility is one of the major complications of Endometriosis, affecting about a third of all cases [2]. The increased risk of infertility adds to the burden of the disease and negatively affects mental health, marital relationships, and social life. Young women with Endometriosis are often concerned that they will not find a life partner willing to accept their condition and potential fertility challenges. Relatedly, some women report that their relationships are affected by the pain, stress, anger, and mood swings caused by Endometriosis [23,33].

Several studies have found negative effects of Endometriosis on psychosocial well-being [23,39,40]. Sims et al. [39] mention that these negative effects can start in early adolescence and include increased emotional and psychosocial distress. Other studies indicate that among women with Endometriosis, there are high rates of anxiety and depression [23,30,40,41], and study participants expressed feelings of worthlessness, guilt, and frustration related to social and relationship limitations arising from their symptoms [42].

Beyond the direct symptoms, managing Endometriosis may also involve addressing lifestyle and behavioral factors that could influence disease severity and life course impact. The pathophysiology of Endometriosis involves estrogens and inflammation. Maintaining a diet low in trans fats, palmitic acid, and red meat can help reduce the risk and pain associated with the condition. Additionally, incorporating fiber, antioxidants, and vitamin D from plant-based foods may alleviate typical symptoms [43]. While studies exploring the link between lifestyle and Endometriosis have not provided definitive evidence to establish whether lifestyle is a cause or the pain influences women’s lifestyles [44,45,46], maintaining a healthy lifestyle has the potential to enhance overall health and assist women with Endometriosis in mitigating the severity of their symptoms and pain [45,47,48]. Studies indicate that proper nutrition and regular physical activity can enhance pain conditions [45,46,49]. Exercise stimulates the release of endorphins, which function as natural pain relievers and may aid in reducing pain [48]. One primary treatment for Endometriosis involves lowering the woman’s estrogen levels [43]. Engaging in regular exercise can decrease estrogen levels in the body, leading to an improvement in Endometriosis symptoms [48].

Unhealthy habits, such as smoking, negatively affect one’s health and are associated with chronic pain [50,51]. However, a meta-analysis combining data from 38 studies found no association between smoking and the risk of Endometriosis [52]. Another review from 2020 also failed to find evidence of a strong scientific link between environmental and lifestyle factors and Endometriosis [52]. Understanding the influence of these lifestyle factors is crucial for developing effective strategies to manage Endometriosis-related pain and improve the quality of life for affected women.

The literature clearly indicates that Endometriosis impacts multiple aspects of women’s life course over time [23]. Fewer studies have examined the role of social support in women’s life course. Furthermore, as far as we know, none have investigated the association between social negativity and PLCI among women with Endometriosis. This study aims to fill that gap.

Social Support and Social Negativity

As explained above, Endometriosis symptoms significantly affect women’s life course and often require them to receive emotional and instrumental support from partners, families, and friends to be able to cope with them [53,54]. Social support is defined as interpersonal interaction involving one or more of the following: (1) Emotional support, which provides empathy, caring, and concern; (2) instrumental support; (3) feedback, which strengthens the sense of control and self-worth; and (4) informational support, including advice, instructions and useful coping tips [55,56]. Studies have shown that having social support can strengthen one’s ability to cope with stressful life events, leading to better general health and longer life expectancy. It has also been linked to improved well-being, as it helps adapt to and manage stressful situations, reducing anxiety and serving as a protective factor against depression [57,58,59,60]. Moreover, social support enhances self-confidence and fosters a sense of acceptance and inclusion [61,62], and is also an essential resource for helping individuals cope with life adversities caused by pain. Research has shown that people suffering from chronic pain and enjoying the benefit of social support systems experience better functional outcomes than people with limited social interactions [63]. The stress buffering hypothesis suggests that social support can protect individuals during times of stress and prevent or reduce disease symptoms [64]. Social support can serve as a buffer against emotional distress and feelings of loneliness, resulting in improved mental health outcomes [64,65]. Among women with Endometriosis, it can significantly influence their emotional well-being and capacity to cope with the challenges posed by the disease [65]. Additionally, social support is a vital protective factor for individuals with Endometriosis and strengthens their resilience [66]. Schwab et al. [63] discovered that perceived social support is the most significant independent risk factor for resilience among women with Endometriosis, highlighting social networks’ essential role in managing the disease.

Although the topic of social support has been extensively researched, it appears that this issue has not been sufficiently studied among women with Endometriosis, specifically the association of social support on these women’s life course.

At the same time, some studies show the negative impact of social support on health. These studies usually focus on the dearth or absence of social support. A lack of social support refers to the absence of positive social interactions and help from others when needed. This can lead to feelings of isolation and not having anyone to turn to for emotional or practical assistance during challenging times [67]. Such experiences can lead to increased feelings of isolation, lowered self-esteem, and worsening health outcomes for women with Endometriosis [68]. Research results show that poor social support not only contributes to emotional loneliness but can also lead to further mental health issues, such as anxiety and depression, which complicate the disease’s self-management [65].

An additional concept related to negative social support is social negativity, a multidimensional construct encompassing behaviors directed at an individual and perceived as aversive or unwanted. This includes three main dimensions: (1) Conflict: behaviors that provoke disagreements or disputes, such as yelling, arguing, or expressing anger toward the individual. (2) Insensitivity: actions that demonstrate a lack of consideration for an individual’s needs or feelings, such as being unsympathetic or taking advantage of the person. (3) Interference: behaviors that obstruct an individual’s ability to pursue personal goals, including invasion of privacy or making excessive demands [67,69]. Social negativity can have a more immediate and severe negative impact on health outcomes due to its association with stress and physiological dysregulation, such as increased inflammation and elevated blood pressure [70]. Similarly, a lack of social support is also linked to poor health outcomes; however, these consequences may be aligned more with chronic feelings of loneliness and the stress of not having available resources, rather than the direct physiological effects of negative interactions [67,70].

Research indicates that social negativity has a more significant, harmful, and lasting impact on both physical and mental health, as it often undermines anticipated support from social interactions [64]. The presence of social negativity, such as conflict, insensitivity, or interference, may not only fail to buffer stress but also exacerbate it, leading to worse mental health and life outcomes [64]. Recognizing the significant impact of social negativity is essential, as it can greatly diminish the health advantages of social interaction [71,72]. As mentioned above, studies examining the negative effects of social support among women with Endometriosis have focused on the absence or lack of support from friends, family, and/or partners, but did not consider the role of social negativity, as noted by Brooks and Dunkel-Schetter [67].

Understanding the association between social negativity and PLCI could inform more comprehensive care strategies that extend beyond symptom management to include interventions aimed at reducing negative social experiences and enhancing overall quality of life for women with Endometriosis.

The primary hypothesis of this study is that there will be significant differences in PLCI concerning intimacy and relationships, education, and employment between women with Endometriosis and women without the disease. We anticipate that women with Endometriosis will report higher levels of PLCI and lower levels of mental health compared to the control group. Additionally, we expect to find differences in social support, social negativity, and menstrual pain between the two groups; specifically, women with Endometriosis are likely to report higher levels of social negativity and menstrual pain, and lower levels of social support. We also hypothesize that higher levels of social support will be associated with lower PLCI and higher levels of mental health among women with Endometriosis. Similarly, higher levels of social negativity will be associated with higher PLCI and lower levels of mental health. Higher accessibility to healthcare services will be associated with lower PLCI and higher reported mental health.

Therefore, the current study aims to compare the PLCI and mental health outcomes of women with Endometriosis to those without the disease, as well as to explore the personal, social, environmental, and behavioral factors that may be associated with PLCI and mental health among women with Endometriosis.

## 2. Materials and Methods

This cross-sectional study was designed to compare the PLCI and self-reported mental health among women with Endometriosis (N = 270) and women without Endometriosis (N = 273). Data were based on self-reports and collected from 11 February to 21 February 2024.

The current study used an independent response internet panel as a sampling frame (PanelView, Tel Aviv, Israel). Women who were panel members received an invitation via email to participate in a new survey, and those who provided informed consent and met the inclusion criteria were eligible to participate. The inclusion criteria were Jewish women living in Israel, aged 18–50, with and without a diagnosis of Endometriosis. The respondents were divided into the research and control groups using the self-reported question “Have you been diagnosed with Endometriosis based on pathological findings or symptoms identified by a specialist?”.

Women who responded negatively were assigned to the control group. In the group of women with Endometriosis, we included those who have been diagnosed with the disease (either through pathology or by an Endometriosis specialist) as well as women who are currently undergoing the diagnosis process.

It should be noted that of the women with Endometriosis, 149 (55.2%) have already been diagnosed, and 121 women (44.8%) were in the process of being diagnosed. No demographic differences were found between these two subgroups except for age: women who have already been diagnosed were slightly older (M = 34.47, SD = 7.71) than women who were in the process of being diagnosed (M = 31.17, SD = 7.73) (t(268) = 3.49, *p* < 0.001); therefore, we decided to include both sub-groups in the research group.

This study was approved by the Max Stern Yezreel Valley College Ethics Committee, approval no. YVC EMEK 2023-76

### 2.1. Participants

Participants in this study were 543 Israeli women, 270 of whom had Endometriosis (49.7%) and 273 women who did not (50.3%). They were, on average, in their early thirties, mostly married or in a meaningful relationship, with no group differences. Approximately two-thirds of the respondents (63.5%) had an academic education, and most were employed. Approximately half reported a below-average income, another quarter an average income, and another quarter above-average income. A little over half of the respondents were secular, to a slightly higher extent among those with Endometriosis.

Sample size was evaluated using G*Power 3.1 [73,74]. For an analysis of covariance, with two groups, given α = 0.05, a moderate-low effect size *f* = 0.15, and power of 0.85, the resulting sample size was 401 participants. For a multiple regression analysis with 15 predictors, given α = 0.05, a moderate-low effect size *f*^2^ = 0.10, and power of 0.85, the resulting sample size was 222 participants. Fifteen predictors were chosen in order to allow for flexibility in the models. The final number of predictors was lower.

### 2.2. Instruments

The dependent variables in the study were Perceived Life Course Impact (PLCI) [75] and mental health [76,77]. The major independent variables included social support [78,79,80], social negativity [67,69], levels of regular and worst pain, and driving time to a women’s health clinic (see Table 1; for the complete list of variables, refer to the Supplementary Materials).

### 2.3. Data Analysis

Data were analyzed with SPSS ver. 29. Descriptive statistics were used for participants’ demographic and background characteristics, comparing the groups with Z ratios for differences between independent proportions, Chi-square tests, and *t*-tests. Cronbach’s α was used for internal consistency of the scales. The study variables were described with means and standard deviations, and Pearson’s correlations were calculated among them. Distribution normality was assessed with skewness values: positive skewness values were identified for two of the PLCI variables: employment (skewness = 1.03, SE = 0.11) and education (skewness = 1.02, SE = 0.122), as well as for social negativity (skewness = 0.90, SE = 0.10), thus log-transforming them. These transformed variables were used in all final models. All other variables had reasonable skewness values (−0.83 to 0.83, SE = 0.10 to 0.11), and mathematical transformations did not assist in improving them. Associations between the demographic and background variables and the dependent variables were examined with Pearson’s correlations and t-tests in an effort to identify the variables that should be controlled for when examining the study hypotheses. Group differences in the study variables were examined with analyses of covariance. Multiple regression models were calculated for PLCI and mental health, with group, background demographic variables, and study variables. Highest VIF value across the regression models was 1.45, suggesting no collinearity. Further, testing the Mahalanobis distances for multivariate normal distribution revealed that no major deviation from multivariate normality was detected. Durbin tests across the regression models ranged between 1.96 and 2.12, suggesting that the residual was not autocorrelated, and the scatterplots of the standardized residuals against the standardized predicted value suggested that the data were homoscedastic. A sensitivity analysis was conducted for the group differences and multiple regressions, excluding the women who were in the process of being diagnosed. Similar results were found.

## 3. Results

The results of this study reveal some significant differences between women with and without Endometriosis across several health and lifestyle variables, including general health, menstrual-related symptoms, and physical activity. Table 2 presents data regarding the total sample and compares the two groups. Women with Endometriosis reported poorer health than women without Endometriosis, as well as a higher prevalence of additional chronic illnesses (25% vs. 17%). A higher percentage of women with Endometriosis were engaged in physical activity (54%) than the control group (44%), and smoking was overall limited, with no group differences. Menstrual pain was perceived as higher among women with Endometriosis, and while over half to 70% of them reported experiencing changes in their urinary and digestive systems during their period, this was also true for 22–45% of the control group. Furthermore, driving time to a women’s health clinic was longer for the women with Endometriosis than for the control group.

Women with Endometriosis reported the age of symptom onset at about 22 years on average and a diagnosis age of about five years later. The highest pain level was evaluated as eight out of ten, and the general pain level was about five. Over a third reported suffering from Endo belly, about half have changed their diet, and about 44% have tried alternative treatments. For most participants (about 80%), driving time to the Endometriosis clinic was at least half an hour.

Significant correlations (Table 3) were found among the study variables. The three PLCI were positively associated with each other and negatively associated with the participants’ mental health. Further, lower social support, higher social negativity, and higher menstrual pain were associated with greater perceived negative impact in one’s life course. Higher social support, lower social negativity, and lower menstrual pain were associated with better mental health.

Associations between the demographic and background variables and the dependent variables were examined to identify those variables that should be controlled for when examining the study hypotheses. Age was negatively associated with PLCI educational changes (r = −0.12, *p* = 0.018) and with mental health (r = −0.10, *p* = 0.019). Partly religious and religious participants had better mental health than secular ones (r = 0.18, *p* < 0.001). Further, having a chronic illness was positively associated with all PLCI domains (r = 0.12 to r = 0.17, *p* < 0.001) and negatively associated with mental health (r = −0.20, *p* < 0.001). In addition, smoking was positively associated with all PLCI (r = 0.15 to r = 0.18, *p* < 0.001) and negatively associated with mental health (r = −0.13, *p* = 0.003). Lastly, driving time to a women’s health clinic was positively associated with all PLCI domains (r = 0.19 to r = 0.26, *p* < 0.001) and negatively associated with mental health (r = −0.20, *p* < 0.001). Other variables, such as levels of education, income, or physical activity, were not associated with the dependent variables (*p* = 0.066 to *p* = 0.869). Additional background variables were highly dependent on group definition, such as the perception of health, or perceived changes in the urinary or digestive systems during one’s period. Thus, the study hypotheses were examined while controlling for age, level of religiosity (1—secular; 0—partly religious or religious), suffering from a chronic illness (1—yes; 0—no), smoking (1—yes; 0—no), and driving time to a women’s health clinic (1—over half an hour; 0—up to half an hour).

Differences in the study variables by group were examined with analyses of covariance, controlling for age, level of religiosity, having a chronic illness, smoking, and driving time to a women’s health clinic (Table 4). Results show significant group differences. Women with Endometriosis evaluated their PLCI as higher than the control group in all three aspects: intimate (39.41 vs. 18.44, F(1, 498) = 91.08, *p* < 0.001, η^2^ = 0.155), employment (32.58 vs. 19.36, F(1, 506) = 20.75, *p* < 0.001, η^2^ = 0.039), and education (32.75 vs. 16.97, F(1, 390) = 24.92, *p* < 0.001, η^2^ = 0.060), as well as whether their mental health was lower than the control group (3.18 vs. 3.66, F(1, 536) = 22.12, *p* < 0.001, η^2^ = 0.040).

Further, women with Endometriosis described higher menstrual pain, higher social negativity, and lower social support than the control group.

Finally, multiple regression models were calculated for PLCI and mental health with group, the background demographic variables, menstrual pain, social support, and social negativity (Table 5). The effects of the October 7 war were entered as an additional independent variable regarding mental health.

It should be noted that similar differences were found when excluding women who were still in the diagnostic process: PLCI: intimate relationships (*p* < 0.001, η^2^ = 0.153); PLCI: employment (*p* = 0.001, η^2^ = 0.026); PLCI: education (*p* < 0.001, η^2^ = 0.038); mental health (*p* = 0.004, η^2^ = 0.020); social support (*p* = 0.033, η^2^ = 0.011); social negativity (*p* = 0.010, η^2^ = 0.016); and menstrual pain (*p* < 0.001, η^2^ = 0.181).

Results show that all four models are significant. Thirty-nine percent of the variance in PLCI in intimate relationships was explained. A greater perceived negative impact in intimate relationships was reported among women with Endometriosis; women with a chronic illness; women who drive over half an hour to receive women’s health care services; and women with higher menstrual pain, lower social support, and higher social negativity.

Further, 27% of the variance in PLCI in employment was explained by the model. A greater perceived negative impact on employment was reported among women with a chronic illness, higher menstrual pain, lower social support, and higher social negativity.

In addition, 35% of the variance in PLCI in education was explained by the model. A greater perceived negative impact on education was reported among women with Endometriosis; younger women; women with a chronic illness; women who drive over half an hour to receive women’s health care services; and women with higher menstrual pain, lower social support, and higher social negativity.

Finally, 26% of the variance in mental health was explained by the model. Better mental health was reported among partly religious or religious women; women with no chronic illness; women who drive less than half an hour to receive women’s health care services; women who perceive fewer personal effects of the October 7 war; and women with lower menstrual pain, higher social support, and lower social negativity.

It should be noted that similar regression results were found for Table 5 when excluding women who were still in the diagnostic process: PLCI: intimate relationships (Adj. R^2^ = 0.39, *p* < 0.001); PLCI: employment (Adj. R^2^ = 0.26, *p* < 0.001); PLCI: education (Adj. R^2^ = 0.32, *p* < 0.001); and mental health (Adj. R^2^ = 0.27, *p* < 0.001).

Multiple regression models were calculated for PLCI and mental health for women with Endometriosis. Background demographic variables, menstrual pain, social support, and social negativity were entered (Table 6). The effects of the October 7 war were entered as an additional independent variable regarding mental health.

Results show that all four models are significant. Twenty-five percent of the variance in PLCI in intimate relationships was explained. A greater perceived negative impact on intimate relationships was reported by women who drive over half an hour to receive women’s health care services, with higher menstrual pain, lower social support, and higher social negativity.

Further, 26% of the variance in PLCI on employment was explained in the model. A greater perceived negative impact on employment was reported with higher menstrual pain, lower social support, and higher social negativity.

In addition, 35% of the variance in PLCI in education was explained in the model. A greater perceived negative impact relating to education was reported among younger women; women with a chronic illness; women who drive over half an hour to receive women’s health care services; and women with higher menstrual pain, lower social support, and higher social negativity.

Finally, 26% of the variance in mental health was explained in the model. Better mental health was reported among women without chronic illness, women who drive less than half an hour to receive women’s health care services, and women with lower menstrual pain and higher social support. It should be noted that similar regression results were found for Table 6 when excluding women who were still in the diagnostic process: PLCI: intimate relationships (Adj. R^2^ = 0.28, *p* < 0.001); PLCI: employment (Adj. R^2^ = 0.28, *p* < 0.001); PLCI: education (Adj. R^2^ = 0.39, *p* < 0.001); and mental health (Adj. R^2^ = 0.24, *p* < 0.001).

Women with Endometriosis scored significantly higher on all three components of social negativity than the control group (Table 7).

Positive significant correlations were found between the components of social negativity and PLCI in intimate relationships, employment, and education, and negative significant correlations were found between the components of social negativity and mental health. The extent of the correlations regarding PLCI in intimate relationships, employment, and education did not differ between the two groups of women. However, the correlations with mental health ranged between r = −0.35 and r = −0.43 (*p* < 0.001) in the control group and between r = −0.02 (*p* = 0.759) and r = −0.22 (*p* < 0.001) among women with Endometriosis, being significantly higher in the control group (conflict: Z = 3.11 *p* = 0.002,; insensitivity: Z = 2.78, *p* = 0.005; interference Z = 3.97, *p* < 0.001).

## 4. Discussion

The current study aimed to compare the Perceived Life Course Impact (PLCI) and mental health of women with Endometriosis to those without the disease, as well as to explore the personal, behavioral, social, and environmental factors that may be associated with PLCI and mental health among Endometriosis patients. The findings confirm the research hypotheses, showing that women with Endometriosis reported a significantly greater negative PLCI on intimacy and relationships, employment and education, as well as poorer mental health, as compared to the control group. They also reported higher social negativity and lower social support. Based on the current cross-sectional data, social negativity was found as a strong predictor of PLCI, with social support, menstrual pain, and healthcare accessibility also identified as significant predictors.

### 4.1. Perceived Life Course Impact

Women with Endometriosis reported greater negative PLCI on their intimate relationships than women without the disease. These results align with findings from other studies regarding the impact Endometriosis has on women’s intimate relationships [21,33]. Symptoms such as dyspareunia, chronic pelvic pain, fatigue, infertility, depression, and anxiety can lower self-esteem and harm sexual health [21,35,38]. Additionally, some women fear losing their partner because of dyspareunia [33]. All these factors contribute to young women being concerned that their condition may hinder their ability to find a partner, as Endometriosis-related pain, stress, and mood swings often disrupt relationships [23]. These fears, concerns, and pain related to intimate relationships should be addressed in counseling by a multidisciplinary team [33,38].

In terms of education, our results show a significant difference between women with Endometriosis and those without the disease. Although women with Endometriosis in this study were more likely to have an academic education, they reported a higher PLCI on their education than women without the disease. This is supported by the literature showing that Endometriosis influences education, starting in school and continuing on to higher education [23]. Students with Endometriosis deal with physical pain and stress related to attendance and assignment deadlines [23,29]. Education systems should acknowledge that Endometriosis is like other chronic diseases or disabilities, and the students who suffer from it should receive all the support they need from their peers, staff, and faculty, in addition to being allowed to attend class remotely and/or have flexible assignment deadlines [81].

The current study also examined the PLCI of Endometriosis in terms of employment. While the findings show that there are no significant differences in employment rates between women with and without Endometriosis as reported by the participants, women with Endometriosis reported significantly greater PLCI, perceiving a great impact of the disease on their employment status. These findings may suggest that while the disease is not linked to a total lack of employment, it may be associated with difficulties in achieving goals and advancing in one’s career. According to the scientific literature, Endometriosis is linked to a loss of working hours, reduced productivity, delays in professional development, choices of part-time jobs, and lower wages [23,29,30,31]. Employers should understand these women’s needs and allow them to work flexible hours or even provide remote access from home, which can help women with Endometriosis achieve their life goals [37].

Our findings show that women with Endometriosis reported a greater PLCI on their education and employment; however, they were more likely to have an academic education, and, as mentioned above, no significant differences were found in employment status between the two groups. This may be explained by the Selection, Optimization, and Compensation (SOC) model [82]. The SOC explains how individuals adapt to challenges and limitations to achieve their goals despite adversity. Selection refers to women with Endometriosis choosing attainable educational goals and jobs that align with their abilities to cope and succeed. Optimization involves the strategic allocation and enhancement of available resources to maximize educational achievement and work productivity despite health-related challenges. Compensation refers to how women might develop alternative strategies to overcome the limitations and challenges associated with Endometriosis [30,82].

These results are consistent with findings from other studies, which indicate that Endometriosis has multifaceted effects that can significantly impact a woman’s life over time. Studies have shown that it can hinder educational achievement, reduce work productivity, influence career decisions, and place a strain on personal relationships. These combined effects can substantially change the life trajectory for women living with Endometriosis [21,23,29,30,31,33] and emphasize the impact that it has on women’s life course. Understanding the effects of Endometriosis beyond its physical symptoms is an initial step toward supporting women in achieving their full life potential.

### 4.2. Mental Health

Our study also shows that women with Endometriosis have lower levels of mental health as compared to those without the disease. These findings were supported by previous studies showing that women with Endometriosis have mental health issues, including increased emotional and psychosocial distress, anxiety, and depression [23,39,40,83].

Research consistently demonstrates that Endometriosis negatively impacts mental health and quality of life due to chronic and cyclical pelvic pain. Multiple studies, both quantitative and qualitative, confirm this association [83,84,85,86,87]. The current study supports these findings, showing that the differences in mental health scores reported between the two groups are not explained by the group (Endometriosis), but rather by the reported pain levels. Since pain is a well-known symptom of Endometriosis [2], the findings of the current study suggest that the subjective experience and intensity of chronic pain may be more critical determinants of psychological distress than the diagnostic status alone.

This relationship between pain and mental well-being must be integrated into treatment approaches for women with Endometriosis, supporting the shift from focusing exclusively on lesions to addressing symptoms such as pain. Recent research further emphasizes that significant, enduring pain is associated with deteriorating psychological health [37,39,40]. The pain resulting from Endometriosis profoundly disrupts women’s mental health, highlighting the importance of comprehensive treatment plans addressing both physical symptoms and psychological well-being.

### 4.3. Social Interaction Factors

The current study highlights the importance of social interaction in women’s PLCI and mental health. These results align with findings from other studies, emphasizing the importance of social support to health [57,58,59,60], and the negative impact social negativity has on it [67,69]. In our sample, differences were found in the levels of social support and social negativity between women with and without Endometriosis, such that women with Endometriosis reported lower levels of social support and higher levels of social negativity than women without the disease.

These reduced levels of social support among women with Endometriosis may be associated with negative health outcomes. Studies have shown that social support can enhance one’s capacity to handle stressful life events, resulting in improved overall health and increased life expectancy, and a dearth or absence of social support can have significant consequences on health [57,58,59,60]. Since perceived social support reflects an individual’s perception, there can sometimes be a gap between the actual quantity or quality of support received and how it is perceived [88]. Additionally, perceived social support may be linked to the extent of social networks; therefore, women with Endometriosis might be at a disadvantage [63] because the symptoms may impact their social lives [23]. Consequently, increasing support for women with Endometriosis from partners, family, and/or friends can aid them in coping with the disease symptoms.

In contrast to insufficient social support, social negativity actively harms well-being by increasing stress and disrupting expectations in interpersonal relationships [67,89]. Researchers use the term social negativity to describe supportive behaviors directed toward a recipient perceived by that individual as negative, condescending, or unwanted [67]. Thus, social negativity is associated with increased stress and direct physiological consequences that may profoundly affect health outcomes [67,70]. Furthermore, social negativity functions as a stressor, which may have a more substantial impact on health than positive social support [67]. Individuals exposed to social negativity may experience feelings of isolation and lack of understanding, and such negativity may lead to increased levels of psychological stress and distress [67,90]. Research suggests that negative interactions with others can elicit negative emotional responses, distress [68], and biological responses that increase the risk of developing adverse health conditions [91]. Furthermore, negative interactions have been found to increase the likelihood of experiencing mood and anxiety disorders [92].

Both types of social interactions matter, but social negativity often exerts a stronger, harmful influence on mental health, as compared to the positive effects of social support [93,94]. Research indicates that negative interactions are less frequent but more significant than positive support, which aligns with expectations. Additionally, negative interactions may undermine personal control or self-esteem and could trigger adverse mental health responses [95,96].

The current study findings indicate that social support and social negativity are significantly associated with PLCI among women with Endometriosis. This may align with the stress-buffering hypothesis, suggesting that social support can shield individuals from the negative impact of stress [67]. Women with Endometriosis often experience stress due to chronic pain, delayed diagnosis, unsuccessful treatments, and other factors [45], and their condition frequently necessitates support from others [53,54]. Since our findings suggest that social negativity is a major predictor of adverse PLCI and mental health among women with Endometriosis in the current sample, and since the literature shows that social negativity can independently and powerfully exacerbate depressive symptoms [93], the current findings may point to the crucial role of reducing social negativity in moderating PLCI. This hypothesis may require future research examining the role of social negativity in moderating the effect of stress on PLCI and the mental health of women with Endometriosis.

Another notable distinction, deemed significant, highlights the inequality in access to healthcare, specifically the considerable distance women with Endometriosis must traverse to consult a gynecologist. These findings underscore the disparities in healthcare accessibility faced by women suffering from Endometriosis. The healthcare system must focus attentively on populations disproportionately impacted by health inequities [97]. Efforts should be directed toward mitigating these disparities through the establishment of additional clinics specializing in Endometriosis, the integration of specialist physicians into regional healthcare facilities, and the enhancement of clinic availability for affected women.

### 4.4. Behavioral Factors

Behavioral factors examined in this study show evidence that women with Endometriosis engage in more physical activity than those without the condition. These results can be explained by the fact that one of the non-medical alternative treatments for women with Endometriosis is physical activity. The therapeutic effect of physical activity has been known to assist women in reducing pain and stress and improving their quality of life [98,99].

In the current sample, women with Endometriosis reported smoking more than women without the disease. Although these differences were not statistically significant, they were close to being so. This trend may be due to the misconception that tobacco smoking can help reduce stress and alleviate other symptoms related to mental health [100].

### 4.5. Study Limitations

The primary study limitation relates to the selection of participants. Israel is a diverse country where people from various cultures and religions coexist. This study focused on Jewish women who were members of an internet panel and agreed to participate. Unfortunately, this study did not examine the cultural variations among Jewish women, which is an area for future research. Another limitation relates to the research methodology. The cross-sectional nature of this study did not allow for causal examination, which requires a longitudinal design. A follow-up study should examine the disease’s influence across different life periods.

Another limitation is that the participants’ mental health measurement may have been influenced by the events occurring in Israel at the time of the study. During this study, the October 7 War broke out in Israel, which resulted in the postponement of data collection until February 2024. To reduce bias in estimating the participants’ mental health, the research questionnaire included questions assessing their personal resilience and examining whether they had been evacuated from their homes, recruited for combat, or had household members injured during the war. Additionally, the questionnaire needed to be adjusted so that women without Endometriosis can be included in it. The questions in the EIQ [66] were designed for women with Endometriosis in an effort to examine the impact the disease has on their life course; therefore, we needed to change some questions that could potentially introduce bias into the data collection process.

The findings underscore the necessity for forthcoming research to thoroughly investigate the underlying causes of the observed deficiency in social support, as well as the elevated levels of social negativity encountered by women diagnosed with Endometriosis, and to examine the role of social negativity in moderating the effect of stress on the life course and mental health of women with Endometriosis. In addition, research should include these women’s partners, and it is imperative to analyze both the received and perceived types of social support. Furthermore, studies ought to be structured not solely based on self-reporting methods but also on the integration of data sourced from health services, thereby augmenting the accuracy of research outcomes regarding health service utilization, associated costs, and related factors. Lastly, considering that Israel is a multicultural nation, it is essential for future studies to investigate the cultural dimensions relevant to this issue as well.

## 5. Conclusions

Women with Endometriosis perceive the disease as having a significant impact on their lives in aspects of intimacy and relationships, employment, and education. In addition, they report poorer mental health compared to women without the disease.

The current study is among the first to explicitly use Perceived Life Course Impact (PLCI) to assess how women with Endometriosis perceive the cumulative effects of the disease across multiple life domains. By introducing PLCI as a central measure, this study emphasizes the importance of understanding how women with Endometriosis perceive the disease’s impact on their life course.

Three principal findings emerged from the analysis. First, social negativity exhibited stronger associations with PLCI than did social support, suggesting that the absence of negative social interactions may be more critical than the presence of positive ones. Second, healthcare accessibility represented a substantial barrier. Participants were frequently required to travel considerable distances to access specialized Endometriosis care. Third, pain severity, rather than the disease presence per se, demonstrated the strongest association with mental health outcomes, indicating that symptom burden may be more predictive of well-being than diagnostic status alone.

These findings have important clinical implications. Comprehensive care models for women with Endometriosis should incorporate multidisciplinary approaches addressing both medical symptom management and psychosocial determinants of health outcomes. Interventions should focus on improving healthcare accessibility, enhancing social support networks, and implementing targeted strategies to mitigate social negativity within patients’ interpersonal environments. The robust association between social context and life outcomes underscores the necessity for healthcare providers to consider the broader social determinants affecting women’s management of this chronic condition. Future longitudinal research should establish causal relationships and guide psychosocial interventions.

## Figures and Tables

**Table 1:** A summary table of instruments.

Variable	No. of Items	Scale	Total Score	Internal Consistency α
**Dependent**:				
Perceived Life Course Impact (PLCI) refers to women’s self-reports of subjective perceptions of the impact of Endometriosis on their life course across several life domains. This variable was measured using the Endometriosis Impact Questionnaire (EIQ) [75].				
PLCI: intimate relationships [75]	6	0–4 (‘not at all’ to ‘a lot’)	0–100 A higher score represents greater PLCI	0.87
PLCI: employment [75]	8	0–4 (‘not at all’ to ‘a lot’)	0–100 A higher score represents greater PLCI	0.91
PLCI: education [75]	5	0–4 (‘not at all’ to ‘a lot’)	0–100 A higher score represents greater PLCI	0.92
Mental health refers to women’s self-reports on the mental state level. This variable was measured using four items from the 12-item Short Form Health Survey (SF12) [76,77]				
Mental health [76,77]	4	1–6 (‘never’ to ‘all the time’)	1–6 A higher score represents better mental health	0.75
**Independent**:				
Social support [78,79,80]	17	1–7 (‘disagree to a very large extent’, to ‘agree to a very large extent’)	1–7 A higher score represents a greater social support	0.92
Social negativity [67,69] conflict, insensitivity interference	19	1–5 (‘not at all’ to ‘to a very large extent’)	1–5 A higher score represents higher social negativity	0.97
Additional characteristics pertaining to Endometriosis		Age of noticing symptoms; age of diagnosis; use of alternative treatments; has changed her diet; suffers from Endo belly; level of regular and worst pain		
Additional characteristics pertaining to women’s health		Driving time to a women’s health clinic; level of menstrual pain; age of first period; diagnosis of any chronic disease; use of hormone-based birth control devices; experienced changes in the urinary system/digestive system during period; smokes; regular physical activity; pregnancy ever; general health		
Effects of the October 7 war	7	0–1 (no/yes)	0–7 Higher score—greater exposure	
Demographic characteristics		Age, marital status, level of education, employment, income, and level of religiosity		

**Table 2:** Demographic characteristics and health-related characteristics by group (N = 543).

	Categories	Total	Control Group (n = 273)	Women with Endometriosis (n = 270)	Difference
Age, M(SD), range		32.88 (7.97), 18–49	32.77 (8.07)	32.99 (7.88)	t(541) = 0.33 (*p* = 0.744)
Marital status, n (%)	Single	133 (24.5)	60 (22.0)	73 (27.0)	χ^2^(2) = 1.88 (*p* = 0.391)
	Married, in a relationship	385 (70.9)	200 (73.3)	185 (68.5)	
	Divorced, widowed	25 (4.6)	13 (4.7)	12 (4.5)	
Level of education, n (%)	High school, non-academic	198 (36.5)	111 (40.7)	87 (32.2)	Z = 2.04 (*p* = 0.041)
	Academic	345 (63.5)	162 (59.3)	183 (67.8)	
Employment, n (%)	Yes	458 (84.3)	225 (82.4)	233 (86.3)	Z = 1.24 (*p* = 0.214)
Income, n (%)	Below average	276 (50.8)	150 (54.9)	126 (46.7)	χ^2^(2) = 5.15 (*p* = 0.076)
	Average	143 (26.3)	61 (22.3)	82 (30.4)	
	Above average	124 (22.8)	62 (22.7)	62 (23.0)	
Level of religiosity, n (%)	Secular	295 (54.3)	133 (48.7)	162 (60.0)	χ^2^(2) = 8.10 (*p* = 0.017)
	Partly religious	142 (26.2)	76 (27.8)	66 (24.4)	
	Religious	106 (19.5)	64 (23.4)	42 (15.6)	
**Health-related characteristics by groups**					
General health, n (%)	Excellent, very good	317 (58.4)	190 (69.6)	127 (47.0)	χ^2^(2) = 36.78 (*p* < 0.001)
	Good	191 (35.2)	78 (28.6)	113 (41.9)	
	Not so good, poor	35 (6.4)	5 (1.8)	30 (11.1)	
Chronic illness, n (%)	Yes	114 (21.0)	47 (17.2)	67 (24.8)	Z = 2.17 (*p* = 0.030)
Physical activity, n (%)	Yes	266 (49.0)	119 (43.6)	147 (54.4)	Z = 2.53 (*p* = 0.011)
Smoking, n (%)	Yes	117 (21.5)	50 (18.3)	67 (24.8)	Z = 1.84 (*p* = 0.065)
Age of first period, n (%)	9–10	35 (6.4)	15 (5.5)	20 (7.4)	χ^2^(3) = 2.37 (*p* = 0.500)
	11–12	276 (50.8)	135 (49.5)	141 (52.2)	
	13–14	174 (32.0)	95 (34.8)	79 (29.3)	
	15+	58 (10.7)	28 (10.3)	30 (11.1)	
Ever pregnant, n (%)	Yes	319 (58.7)	173 (63.4)	146 (54.1)	Z = 2.20 (*p* = 0.028)
Uses hormonal therapy, n (%)	Yes	338 (62.2)	171 (62.6)	167 (61.9)	Z = 0.19 (*p* = 0.850)
Menstrual pain, M(SD), range		6.41 (2.53), 0–10	5.21 (2.54)	7.61 (1.85)	t(497.36) = 12.60 (*p* < 0.001)
Experiences changes in urinary system during period, n (%)	Yes	200 (36.8)	59 (21.6)	141 (52.2)	χ^2^(2) = 67.21 (*p* < 0.001)
	No	215 (39.6)	150 (54.9)	65 (24.1)	
	Sometimes	128 (23.6)	64 (23.4)	64 (23.7)	
Experiences changes in digestive system during period, n (%)	Yes	307 (56.5)	122 (44.7)	185 (68.5)	χ^2^(2) = 34.26 (*p* < 0.001)
	No	156 (28.7)	106 (38.8)	50 (18.5)	
	Sometimes	80 (14.7)	45 (16.5)	35 (13.0)	
Driving time to a women’s health clinic, n (%)	Up to half an hour	390 (72.1)	225 (83.0)	165 (61.1)	χ^2^(2) = 37.64 (*p* < 0.001)
	Half an hour to an hour	123 (22.7)	43 (15.9)	80 (29.6)	
	Over an hour	28 (5.2)	3 (1.1)	25 (9.3)	
**Health-related characteristics: Women with Endometriosis** (N = 270)					
Age of first symptoms, M(SD), range		-	-	21.67 (8.25), 7–48	
Age of diagnosis, M(SD), range		-	-	27.07 (7.76), 9–48	
Worst pain during Endo, M(SD), range		-	-	8.17 (1.93), 0–10	
General pain during Endo, M(SD), range		-	-	4.93 (2.50), 0–10	
Suffers from Endo belly, n (%)	Yes	-	-	102 (37.8)	
Used alternative treatment, n (%)	Yes	-	-	120 (44.4)	
Changed diet, n (%)	Yes	-	-	137 (50.7)	
Driving time to an Endometriosis clinic, n (%)	Up to half an hour	-	-	52 (21.1)	
	Half an hour to an hour	-	-	110 (44.7)	
	Over an hour	-	-	84 (34.2)	

**Table 3:** Means, standard deviations, and correlations for the study variables (N = 543).

	M(SD)	1.	2.	3.	4.	5.	6.	7.
1. Life course: intimate relationships (n = 505)	29.15 (23.70)	1						
2. Life course: employment (n = 513)	26.10 (23.92)	0.47 *	1					
3. Life course: education (n = 397)	25.28 (27.84)	0.51 *	0.71 *	1				
4. Mental health	3.42 (0.92)	−0.41 *	−0.46 *	−0.40 *	1			
5. Social support	5.30 (1.28)	−0.37 *	−0.34 *	−0.38 *	0.35 *	1		
6. Social negativity	1.94 (0.92)	0.48 *	0.46 *	0.47 *	−0.35 *	−0.48 *	1	
7. Menstrual pain	6.41 (2.53)	0.28 *	0.21 *	0.23 *	−0.25 *	−0.01	0.05	1

* *p* < 0.001. Note. Range: Life course variables: 0–100; mental health: 1–6; social support: 1–7; social negativity: 1–5; menstrual pain: 1–10.

**Table 4:** Means, standard deviations, and F values for the study variables by group (N = 543).

	Total M (SD)	Control Group (n = 273) M (SD)	Women with Endometriosis (n = 270) M (SD)	F (df) (*p*) (η^2^)
PLCI: intimate relationships (n = 505)	29.15 (23.70)	18.44 (16.11)	39.41 (25.24)	F(1, 498) = 91.08, (*p* < 0.001) (η^2^ = 0.155)
PLCI: employment (n = 513)	26.10 (23.92)	19.39 (19.12)	32.58 (26.23)	F(1, 506) = 20.75, (*p* < 0.001) (η^2^ = 0.039)
PLCI: education (n = 397)	25.28 (27.84)	16.97 (24.05)	32.75 (28.92)	F(1, 390) = 24.92, (*p* < 0.001) (η^2^ = 0.060)
Mental health	3.42 (0.92)	3.66 (0.90)	3.18 (0.87)	F(1, 536) = 22.12, (*p* < 0.001) (η^2^ = 0.040)
Social support	5.30 (1.28)	5.56 (1.13)	5.04 (1.36)	F(1, 536) = 13.26, (*p* < 0.001) (η^2^ = 0.024)
Social negativity	1.94 (0.92)	1.74 (0.80)	2.13 (0.99)	F(1, 536) = 13.86, (*p* < 0.001) (η^2^ = 0.025)
Menstrual pain	6.41 (2.53)	5.21 (2.54)	7.61 (1.85)	F(1, 536) = 135.50, (*p* < 0.001) (η^2^ = 0.202)

Note. Range: PLCI variables 0–100; mental health 1–6; social support 1–7; social negativity 1–5; menstrual pain 1–10. Differences were calculated with log-transformed variables.

**Table 5:** Multiple regression models for PLCI and mental health.

	PLCI: Intimate Relationships (n = 505) β (*p*)	PLCI: Employment (n = 513) β (*p*)	PLCI: Education (n = 397) β (*p*)	Mental Health (n = 543) β (*p*)
Group (Endometriosis)	0.26 (<0.001)	0.06 (0.170)	0.12 (0.015)	−0.05 (0.303)
Age	−0.02 (0.544)	−0.04 (0.252)	−0.19 (<0.001)	−0.04 (0.270)
Level of religiosity (secular)	−0.01 (0.969)	0.02 (0.561)	0.01 (0.790)	−0.13 (<0.001)
Chronic illness (yes)	0.09 (0.013)	0.04 (0.253)	0.09 (0.030)	−0.12 (0.002)
Smoking (yes)	0.02 (0.575)	0.08 (0.038)	0.04 (0.319)	−0.01 (0.779)
Driving time (over half an hour)	0.10 (0.005)	0.06 (0.108)	0.10 (0.028)	−0.10 (0.015)
Effects of war	--	--	--	−0.08 (0.041)
Menstrual pain	0.11 (0.005)	0.14 (0.002)	0.12 (0.012)	−0.17 (<0.001)
Social support	−0.12 (0.004)	−0.14 (0.002)	−0.19 (<0.001)	0.20 (<0.001)
Social negativity	0.34 (<0.001)	0.35 (<0.001)	0.33 (<0.001)	−0.18 (<0.001)
Adj. R^2^	0.39	0.27	0.35	0.26
*p*	*p* < 0.001	*p* < 0.001	*p* < 0.001	*p* < 0.001

Note. Regression models were calculated with log-transformed variables.

**Table 6:** Multiple regression models for PLCI and mental health in women with Endometriosis.

	PLCI: Intimate Relationships (n = 258) β (*p*)	PLCI: Employment (n = 261) β (*p*)	PLCI: Education (n = 208) β (*p*)	Mental Health (n = 270) β (*p*)
Age	−0.03 (0.610)	0.01 (0.838)	−0.13 (0.031)	0.01 (0.971)
Level of religiosity (secular)	−0.03 (0.658)	−0.07 (0.212)	−0.01 (0.845)	−0.08 (0.170)
Chronic illness (yes)	0.10 (0.065)	0.01 (0.818)	0.12 (0.037)	−0.19 (<0.001)
Smoking (yes)	0.05 (0.400)	0.09 (0.096)	0.08 (0.202)	0.03 (0.563)
Driving time (over half an hour)	0.14 (0.015)	0.10 (0.063)	0.18 (0.002)	−0.14 (0.015)
Effects of war	--	--	--	−0.04 (0.451)
Menstrual pain	0.17 (0.003)	0.18 (0.001)	0.20 (<0.001)	−0.17 (0.003)
Social support	−0.15 (0.013)	−0.20 (0.001)	−0.18 (0.006)	0.26 (<0.001)
Social negativity	0.34 (<0.001)	0.32 (<0.001)	0.37 (<0.001)	−0.04 (0.493)
Adj. R^2^	0.25	0.26	0.35	0.18
*p*	*p* < 0.001	*p* < 0.001	*p* < 0.001	*p* < 0.001

Note. Regression models were calculated with log-transformed variables.

**Table 7:** Group differences in social negativity (N = 543).

	Total M (SD)	Control Group (n = 273) M (SD)	Women with Endometriosis (n = 270) M (SD)	t (df) (*p*)
Conflict	1.97 (0.92)	1.80 (0.82)	2.14 (0.99)	t(522.27) = 4.37 (*p* < 0.001)
Insensitivity	1.98 (1.05)	1.78 (0.95)	2.19 (1.11)	t(526.93) = 4.55 (*p* < 0.001)
Interference	1.78 (0.91)	1.55 (0.75)	2.02 (1.00)	t(497.81) = 6.18 (*p* < 0.001)

Note. Range 1–5.

## Data Availability

Data availability by request from chenz@yvc.ac.il.

## Supplementary Materials

The following supporting information can be downloaded at https://www.mdpi.com/article/10.3390/jcm14134761/s1. Supplementary Material S1. Complete list of research variables and their measurement details. *Dependent Variables*: (1) Life Course Impact—Intimate Relationships: Six items, scale 0–4, α = 0.87, total score 0–100; (2) Life Course Impact—Employment: Eight items, scale 0–4, α = 0.91, total score 0–100; (3) Life Course Impact—Education: Five items, scale 0–4, α = 0.92, total score 0–100; (4) Mental Health: Four items from SF12, scale 1–6, α = 0.75, total score 1–6. *Independent Variables*: (1) Social Support: 17 statements, scale 1–7, α = 0.92, total score 1–7; Social Negativity: 19 questions (3 dimensions), scale 1–5, α = 0.97, total score 1–5; (2) Additional Endometriosis Characteristics: Age of symptoms onset, diagnosis age, alternative treatments, diet changes, endo belly, pain levels (0–10); (3) Additional Women’s Health Characteristics: Driving time to clinic, menstrual pain (0–10), age of menarche, chronic diseases, birth control use, urinal/digestive changes, smoking, physical activity, pregnancy history, general health; (4) October 7 War Effects: Seven dichotomous items, total score 0–7; (5) Demographic Characteristics: Age, marital status, education level, employment, income, religiosity level.
