# Supplementary material for: Understanding the Role of Social Negativity in Perceived Life Course Impact and Mental Health Among Women with Endometriosis
Source: J Clin Med. 2025 Jul 5;14(13):4761. doi: 10.3390/jcm14134761 (PMC12251333; doi:10.3390/jcm14134761)
Supplement: Supplementary file 1 [file jcm-14-04761-s001.zip › jcm-3632003-supplementary.pdf]

Section S2.2 Instruments

S2.2.1 Dependent Variables

Life course impact: This variable was tested through validated questions from the Endometriosis Impact Questionnaire [75]. Life course impact evaluation in the current study included three aspects:

Life course: intimate relationships: Six items on a scale of 0-4 ('not at all' to 'a lot'). α = .87. Total score 0-100. Higher score - greater negative impact. Sample items include: "I had pain during or after sexual activity," and "I experienced tension in my relationship with my partner."

Life course: employment: Eight items on a scale of 0-4 ('not at all' to 'a lot'). α = .91. Total score 0-100. Higher score - greater negative change. Sample items include: "I had to miss work", "I reduced my working hours".

Life course: education: Five items on a scale of 0-4 ('not at all' to 'a lot'). α = .92. Total score 0-100. Higher score - greater negative change. Sample items include: "I had to take time off or stop my studies", "I did not complete my school assignments on time".

Mental health was tested using four items from the 12-item Short Form Health Survey (SF12) – the shortened version of the SF36. The four items, scale 1-6 ('never' to 'all the time'). α = .75. Total score 1-6, a higher score reflecting a more positive state. Sample items include: "In the last four weeks, did you feel peaceful and calm?", "In the last four weeks, how much did your physical health condition or your emotional problems interfere with your social activities (such as visiting friends or relatives)?" [76-77]

2.2.2 Independent Variables

Social interactions contain two aspects - social support and social negativity.

Social support – The women's degree of perceived positive social support were examined with reference to three sources: family, friends and significant others (such as physicians or supervisors at work). To examine this variable, we combined questions from two social support questionnaires. The first is the Multidimensional Scale of Perceived Social Support (α = .85-.91), translated into Hebrew by Statman (Family Scale α = .93; Friends Scale α = .91; Others Scale α = .91). Sample items include: "My family really tries to help me"; "There is a person close to me who is near me when I need him/her". The second questionnaire is the Interpersonal Social Evaluation List (ISEL; α = .88), which examines instrumental and decision-making support; for example, "If I need a ride to the doctor, I will have trouble finding someone to take me (without pay)". This variable was tested using 17 statements, with answers ranging from 1 - disagree to a very large extent, to 7 - agree to a very large extent (α = .92). Total score 1-7, a higher score reflects greater social support [78-80].

Social negativity – This concept was examined through 19 questions addressing three dimensions: conflict (8 items); insensitivity (7); and interference (4). Cronbach's alpha values among Jewish women were 0.95, 0.94, 0.83, and 0.96 for conflicts, insensitivity, interference, and the total scale, respectively. For Arab women, they were 0.94, 0.94, 0.74, and 0.96, respectively. These dimensions were examined on a scale from 1 - not at all to 5 - to a very large extent [67, 69]. (α = .97). Total score 1-5, a higher score reflects greater social negativity.

Additional characteristics pertaining to Endometriosis – Age of noticing symptoms; age of diagnosis; uses alternative treatments (yes/no); has changed her diet (yes/no); suffers from Endo belly (yes/no); level of regular and worst pain (scale 0-10).

Additional characteristics pertaining to women's health - Driving time to a women's health clinic (0-up to half an hour, 1-over half an hour); level of menstrual pain (scale 0-10); age of first period (a- 9-10 years, b- 11-12 years, c- 13-14 years, d- 15+ years); diagnosis of any chronic disease; uses hormone-based birth control devices; experiences changes in the urinal

system/digestive system during period (yes/no/sometimes); smokes (yes/no); regular physical activity (yes/no); pregnancy ever (yes/no); general health (very bad to very good).

Effects of the October 7 war in Israel – Seven dichotomous items pertaining to the respondents' experience with the war: being evacuated from home, a close family member or the respondent were hurt, another family member/friend was hurt, sirens go off in place of living, the respondent is in reserve duty, the respondent's partner is in reserve duty, a close family member is in reserve duty. The total score was composed of the sum of the items, ranging 0-7. This variable was used as a control variable for the model predicting mental health levels.

Demographic characteristics – Age (by years), marital status (a. single, b. married, in a relationship, c. divorced, widowed), level of education (a. high school, non-academic, b. academic), employment (a. yes, b. no), income (a. below average, b. average, c. above average), and level of religiosity (a. secular, b. partly religious, c. religious).
